# Comparison of prognosis and analysis of related risk factors among three different left atrial appendage occlusion procedures in patients with atrial fibrillation

**DOI:** 10.3389/fcvm.2025.1534899

**Published:** 2025-02-17

**Authors:** Xiao-hai Jiang, Yan-juan Tan, Run-zhong Wang, Zhong-bao Ruan, Li Zhu

**Affiliations:** ^1^ Department of Cardiology, The affiliated Taizhou People's Hospital of Nanjing Medical University, Taizhou School of Clinical Medicine, Nanjing Medical University, Taizhou, Jiangsu, China; ^2^ Postgraduate Training Base of Dalian Medical University, Taizhou People's Hospital, Taizhou, Jiangsu, China

**Keywords:** atrial fibrillation, left atrial appendage occlusion, complete occlusion, peri-device leak, device related thrombosis

## Abstract

**Background:**

Left atrial appendage occlusion (LAAO) serves as an alternative to oral anticoagulation (OAC) for atrial fibrillation (AF) patients at high risk of bleeding. The aim of this study was to compare the peri-procedural safety, complete or incomplete occlusion, the incidence of the peri-device leak (PDL), and device-related thrombosis (DRT) among LAAO, cryoballoon ablation (CBA) combined with LAAO, and radiofrequency catheter ablation (RFCA) combined with LAAO and to explore the risk factors of PDL and incomplete occlusion.

**Methods:**

382 patients with non-valvular AF who underwent either LAAO alone (*n* = 117), CBA combined with LAAO (*n* = 125), or RFCA combined with LAAO (*n* = 140) were included in the retrospective study. The study assessed peri-procedural complications and imaging results (3 months post-procedure). Multivariable logistic regression was employed to identify risk factors for incomplete occlusion and PDL.

**Results:**

Peri-procedural complication rates were low among all groups, with 2.9% in the RFCA combined with the LAAO group. In contrast, the LAAO alone and CBA combined with LAAO groups reported no major complications (*p* = 0.347). At the 3-month follow-up, the incidence of DRT was 1.7% in the LAAO group, 2.4% in the CBA combined with the LAAO group, and 2.1% in the RFCA combined with the LAAO group (*p* = 0.930). Complete occlusion rates were comparable among the groups: 64.8% for CBA combined with LAAO, 62.4% for LAAO alone, and 60.7% for RFCA combined with LAAO (*p* = 0.794). PDL occurred in 33.3% of LAAO-alone patients, 34.4% of CBA combined with LAAO patients, and 38.6% of RFCA combined with LAAO patients (*p* = 0.644). Multivariable analysis identified persistent AF and serum creatinine (SCr) as independent predictors of PDL and incomplete occlusion.

**Conclusion:**

Peri-procedural complications, complete occlusion, PDL, and DRT rates were similar across the three treatment strategies. Persistent AF and SCr were significant risk factors for incomplete occlusion and PDL. These findings highlight the importance of individualized treatment strategies based on patient-specific risk factors for optimizing outcomes.

## Graphical Abstract

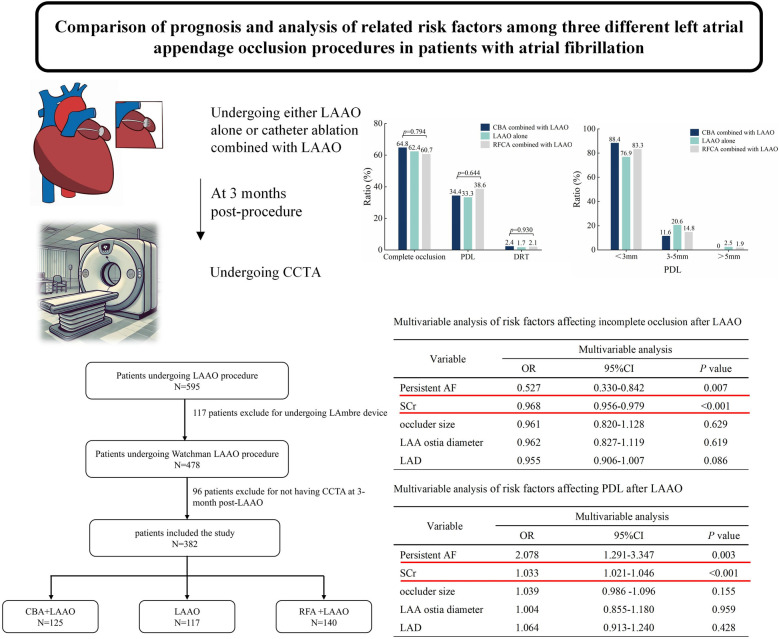

## 1 Introduction

Atrial fibrillation (AF) is a common cardiac arrhythmia, and one of its most severe complications is stroke (1). Thrombus formation is the primary cause of stroke, with over 90% of thrombi in AF patients originating from the left atrial appendage (LAA) (2, 3). Traditionally, oral anticoagulants (OACs) have been the mainstay for stroke prevention in patients with AF. However, the bleeding risk associated with long-term use of anticoagulants has prompted clinicians to explore alternative preventive strategies (4, 5). Consequently, left atrial appendage occlusion (LAAO) has emerged as an effective alternative to anticoagulant therapy for AF patients at high risk of bleeding or those who are unwilling to receive anticoagulant therapy or have contraindications to anticoagulant therapy. Several studies have confirmed its clinical efficacy and safety (6, 7).

LAAO reduces the risk of stroke by mechanically sealing the LAA and has become an important therapeutic option in the management of AF. In addition to LAAO alone, recent attention has been given to a “one-stop” approach combining LAAO with catheter ablation (CA). CA primarily involves two techniques: cryoballoon ablation (CBA) and radiofrequency catheter ablation (RFCA). CBA isolates the pulmonary veins using geothermal energy, while RFCA disrupts abnormal atrial electrical conduction pathways through thermal energy. Existing studies suggest that this combined procedure effectively restores sinus rhythm and prevents thrombus formation without significantly increasing the risk of serious adverse events (SAEs) during the peri-procedural period (8). Although most research has focused on evaluating the safety and efficacy of RFCA combined with LAAO, studies on the clinical outcomes of CBA combined with LAAO remain relatively scarce (9–15). Moreover, only a limited number of comparative studies have investigated the effects of combining LAAO with either RFCA or CBA (16). To date, comparative studies on the efficacy of these three surgical approaches are relatively limited. Therefore, this study aimed to compare the outcomes of three different treatment strategies in patients with AF, focusing on peri-procedural complications and post-procedural imaging results, specifically device-related thrombus (DRT), peri-device leak (PDL) and the rate of complete occlusion. By providing a comparative analysis of these outcomes, this study seeks to offer valuable insights into the individualized treatment of AF patients.

## 2 Method

### 2.1 Study population

**Figure 1:**
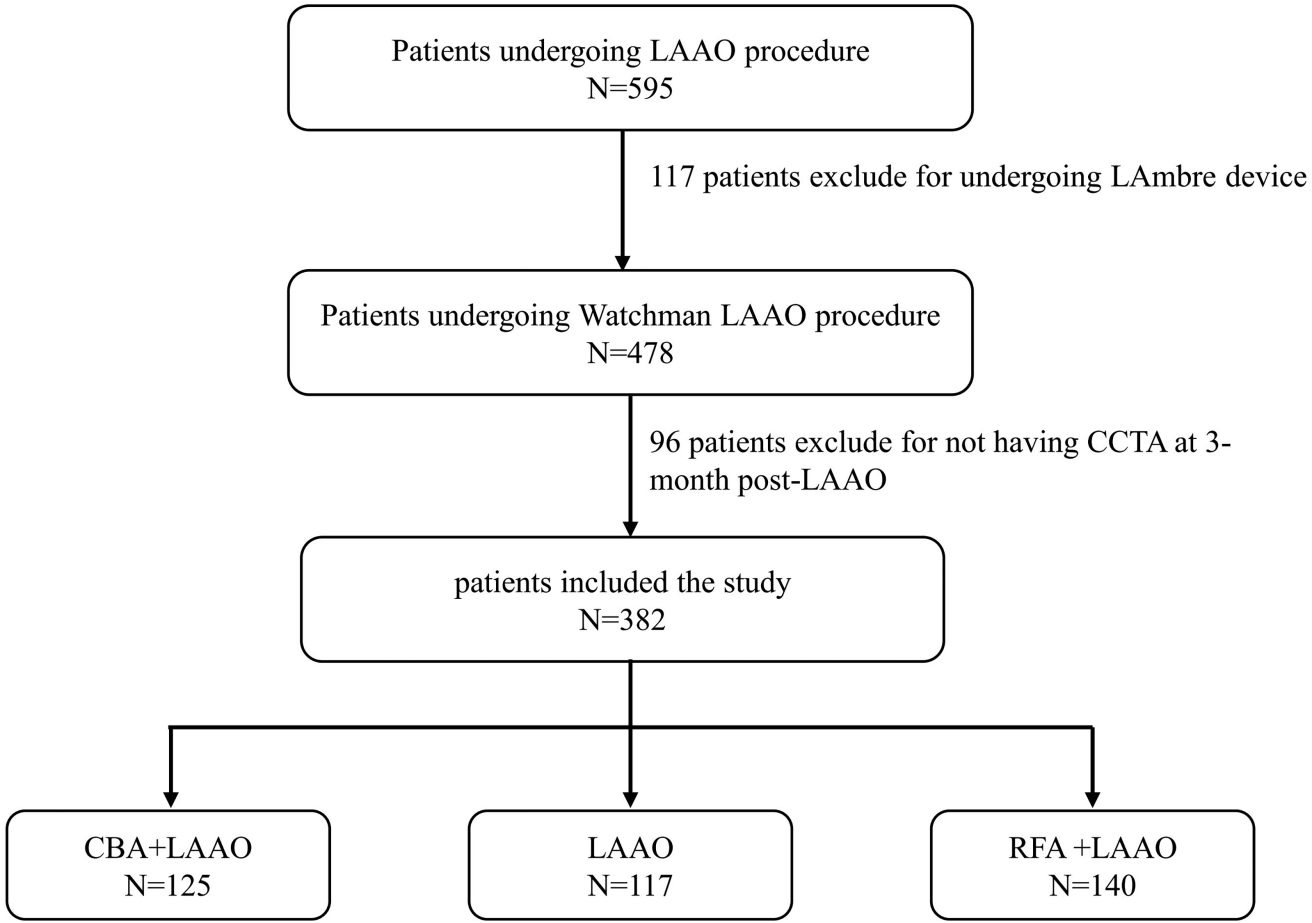
Study flowchart.

This retrospective study included those patients with non-valvular AF who underwent LAAO at the Electrophysiology Center of the affiliated Taizhou People's Hospital of Nanjing Medical University. Patients were included if they were adults with symptomatic non-valvular paroxysmal or persistent AF and were at high risk of stroke (CHA_2_DS_2_-VASc score ≥2 for males, ≥3 for females). Additionally, all patients met at least one of the following criteria: (1) electrical isolation of the pulmonary vein during CA; (2) contraindications to OAC; (3) unwillingness or inability to take OAC; (4) a history of stroke while on OAC. Five hundred ninety-five patients were initially screened, 117 of whom received the LAmbre device. After excluding 96 patients who did not undergo coronary computed tomography angiography (CCTA) at 3 months post-LAAO, 382 patients who performed LAAO with the Watchman2.5 device were included in the final analysis (Figure 1).

### 2.2 Procedure

#### 2.2.1 LAAO alone

As we described previously (17), femoral venous access was established under local or general anesthesia, followed by transseptal puncture to gain entry into the left atrium (LA). Subsequently, heparin was administered, and the activated clotting time (ACT) was meticulously monitored to ensure it remained above 300 s. The anatomical structure of the LAA was evaluated using fluoroscopy to confirm the absence of thrombus formation. Based on the LAA anatomy, the appropriate Watchman occluder was selected and delivered into the LAA via a catheter. The device was then adjusted according to the “PASS” criteria (Position, Anchor, Size, Seal) to ensure optimal placement and effective sealing. After deployment, the occluder's position was confirmed with contrast imaging, ensuring any residual leak was less than 5 mm. If the leak exceeded this threshold, the occluder was repositioned or replaced with a larger device.

#### 2.2.2 RFCA combined with LAAO

Following transseptal puncture, intravenous heparin was administered as a bolus, and the ACT was closely monitored to ensure it remained above 300 s. As our previous report (18), after establishing femoral venous access and performing a transseptal puncture to enter the LA, a mapping catheter was used to perform electroanatomic mapping and identify the target ablation areas. Ablation was performed with a power setting of 35–45 watts for 20–30 s per pulmonary vein until all electrical activity in the pulmonary veins was completely isolated. After ablation, LAAO was immediately performed using the Watchman occluder.

#### 2.2.3 CBA combined with LAAO

CBA was performed through femoral venous access into the LA, followed by administering heparin and close monitoring of the ACT to maintain a value exceeding 300 s. Each pulmonary vein was confirmed using contrast injection via the catheter, and cryoablation was performed for 180–240 s, with temperatures between −45℃ and −55℃. Upon successful ablation, LAAO was performed immediately using the Watchman occluder.

### 2.3 Endpoints

At 3 months post-procedure, all patients underwent CCTA with the Siemens SOMATOM Force 128-slice dual-source CT scanner. A contrast agent was injected at a rate of 4–5 ml/s with 80 ml of non-ionic contrast media to enhance image quality. Scanning parameters were set at 100–120 kV tube voltage and 800–1,235 mA tube current, with a collimation width of 256 × 0.625 mm, 270 ms rotation time, and 0.2 mm slice thickness. Dual-phase scanning was performed, with the second phase initiated 60 s after the first. Radiation dosage was automatically controlled by ECG-triggered prospective gating, with 30% and 80% of the R-R interval exposure. The scanning range extended from 1 cm below the carina to the cardiac diaphragm. All imaging data were processed using the Syngo VB10 workstation (Siemens), with analyses including 3D reconstruction, volume rendering (VR), and maximum intensity projection (MIP)—the procedures described above assessed the incidents of complete or incomplete occlusion, PDL and DRT.

### 2.4 Definitions

**Figure 2:**
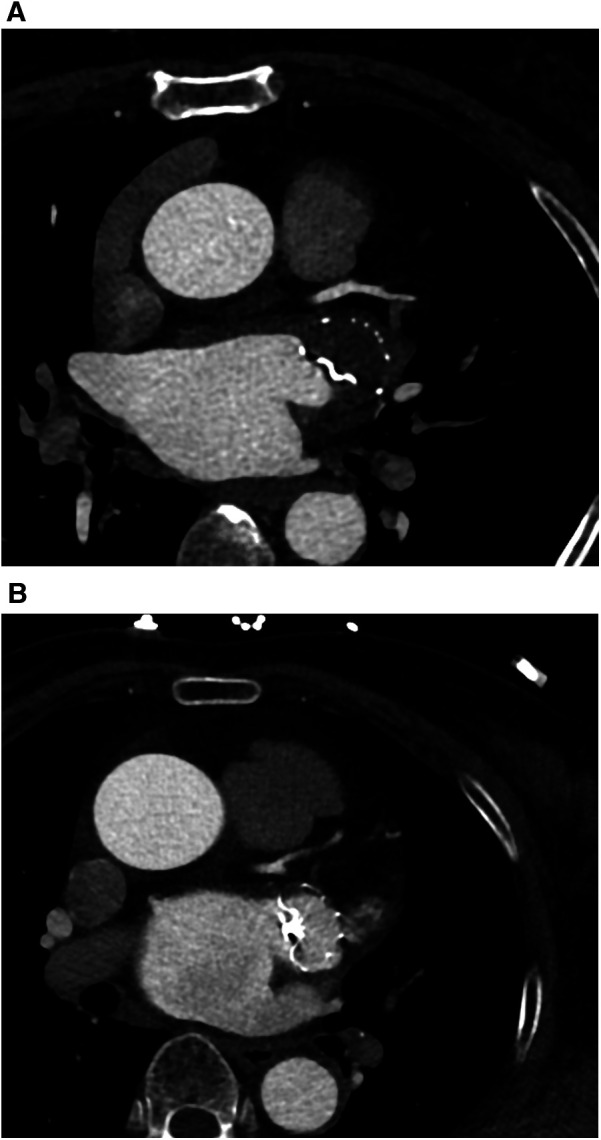
**(A)** One case (a 63-year-old woman with persistent atrial fibrillation) for complete occlusion at 3 months follow-up after LAAO. LAA CTA showed no residual contrast agent flow inside the LAA. **(B)** One case (a 68-year-old man with persistent atrial fibrillation) for incomplete occlusion at 3 months follow-up after LAAO. LAA CTA showed residual contrast agent flow inside the LAA.

In this study, complete occlusion is defined by an LAA attenuation value of <100 HU or an LAA LA attenuation ratio of ≤0.25 (Figure 2A). Incomplete occlusion is characterized by a CT attenuation value >100 HU or an LAA/LA CT attenuation ratio of >0.25 (Figure 2B). Incomplete occlusion includes trans-fabric leaks, PDL, and mixed leaks. Trans-fabric leak refers to contrast leakage through the fabric structure of the device into the LAA, rather than from the edge of the device. PDL refers to contrast leakage around the occlusion device. DRT is defined as thrombus formation on or around the device, typically detected by imaging, and is characterized by nodular or mass-like areas of enhancement defects, with regions of pronounced low attenuation thickening.

### 2.5 Statistical analysis

Continuous variables with a normal distribution were expressed as mean ± standard deviation (SD), while non-normally distributed variables were presented as median and interquartile range (IQR). Categorical variables were described as numbers and percentages. Differences in continuous variables were assessed using the *T*-test or Mann–Whitney *U* test, and differences in categorical variables were analyzed using the Pearson chi-square test or Fisher's exact test. Univariate logistic regression analysis was performed to evaluate the risk factors, with variables showing a *p*-value of <0.05 included in the multivariate analysis. Multivariate logistic regression was used to further adjust for confounding factors. All statistical analyses were conducted using SPSS 27.0 software, and a two-sided *p*-value of <0.05 was considered statistically significant.

## 3 Results

### 3.1 Baseline characteristics

A total of 382 patients who successfully underwent Watchman occluder implantation and completed CCTA three months post-procedure were included in the study. Of these, 125 patients were in the CBA combined with the LAAO group, 117 in the LAAO alone group, and 140 in the RFCA combined with the LAAO group. Baseline characteristics are summarized in Table 1. There were no significant differences in age, sex, AF type, or comorbidities [including heart failure, hypertension, diabetes, coronary artery disease (CAD), prior stroke, vascular disease, abnormal renal function, smoking, or alcohol consumption] among the three groups (*p* > 0.05). Furthermore, no significant differences were found in the CHA_2_DS_2_-VASc score, HAS-BLED score, NT-proBNP levels, left atrial diameter (LAD), left ventricular ejection fraction (LVEF), or anticoagulation therapy regimen among the groups (*p* > 0.05). However, a significant difference was observed in the LAA ostial diameter among the three groups (*p* = 0.006), with a value of 22.4 ± 3.4 mm in the CBA combined with the LAAO group, 23.3 ± 3.5 mm in the RFCA combined with LAAO group, and 23.8 ± 3.7 mm in the LAAO alone group.

**Table 1:** Patient characteristics at baseline.

variable	CBA combined with LAAO (*n* = 125)	LAAO alone (*n* = 117)	RFCA combined with LAAO (*n* = 140)	*P* value
Age, years	69.3 ± 8.3	69.6 ± 7.7	67.6 ± 8.0	0.087
Sex, *n* (%)				0.524
Male	63 (50.4)	67 (57.3)	78 (55.7)	
Female	62 (49.6)	50 (43.1)	62 (44.3)	
BMI, kg/m^2^	24.9 ± 2.9	25.4 ± 3.1	25.5 ± 3.0	0.242
AF type, *n* (%)				0.062
Paroxysmal	61 (48.8)	69 (59.0)	88 (62.9)	
Persistent	64 (51.2)	48 (41.0)	52 (37.1)	
Heart failure, *n* (%)				0.054
Yes	26 (20.8)	39 (33.3)	45 (32.1)	
No	99 (79.2)	78 (66.7)	95 (67.9)	
Hypertension, *n* (%)				0.301
Yes	75 (60.0)	81 (69.2)	93 (66.4)	
No	50 (40.0)	36 (30.8)	47 (33.6)	
Diabetes, *n* (%)				0.212
Yes	28 (22.4)	17 (14.5)	31 (22.1)	
No	97 (77.6)	100 (85.5)	109 (77.9)	
CAD, *n* (%)				0.186
Yes	25 (20.0)	24 (20.5)	18 (12.9)	
No	100 (80.0)	93 (79.5)	122 (87.1)	
Stroke, *n* (%)				0.369
Yes	26 (20.8)	31 (26.5)	27 (19.3)	
No	99 (79.2)	86 (73.5)	113 (80.7)	
Vascular disease, *n* (%)				0.220
Yes	14 (11.2)	7 (6.0)	17 (12.1)	
No	111 (88.8)	110 (94.0)	123 (87.9)	
Abnormal renal function, *n* (%)				0.256
Yes	5 (4.0)	2 (1.7)	8 (5.7)	
No	120 (96)	115 (98.3)	132 (94.3)	
Smoking, *n* (%)				0.060
Yes	21 (16.8)	20 (17.1)	38 (27.1)	
No	101 (83.2)	97 (82.9)	102 (72.9)	
Alcohol drinking, *n* (%)				0.198
Yes	14 (11.2)	12 (10.3)	24 (17.1)	
No	111 (88.8)	105 (89.7)	116 (82.9)	
CHA_2_DS_2_-VASc, score	3 (2, 4)	3 (3, 4)	3 (2, 4)	0.057
HAS-BLED, score	2 (1, 3)	2 (1, 3)	2 (1, 2)	0.210
NT-proBNP, ng/L	866.9 (538.0, 1,702.5)	946.8 (437.6, 1,863.2)	814.7 (441.6, 2,115.3)	0.959
ALT, U/L	27.1 ± 19.1	28.0 ± 27.6	28.7 ± 18.4	0.833
AST, U/L	27.6 ± 12.6	30.6 ± 24.8	29.2 ± 14.9	0.694
SCr, umol/L	74.9 ± 40.2	73.4 ± 18.4	74.7 ± 27.7	0.520
TC, mmol/L	3.7 ± 0.9	3.7 ± 1.0	4.0 ± 1.1	0.064
TG, mmol/L	1.7 ± 1.3	1.6 ± 1.3	1.7 ± 1.6	0.807
LDL-C, mmol/L	2.5 ± 1.2	2.3 ± 1.1	2.7 ± 1.2	0.053
LAD, mm	47.6 ± 4.4	46.0 ± 4.8	46.6 ± 4.7	0.349
LVEF, %	61.7 ± 8.0	59.9 ± 10.4	60.8 ± 9.4	0.562
Occluder size, (*n*, %)				0.161
21 mm	8 (6.4)	4 (3.4)	3 (2.1)	
24 mm	19 (15.2)	13 (11.1)	19 (13.6)	
27 mm	42 (33.6)	26 (22.2)	36 (25.7)	
30 mm	31 (24.8)	34 (29.1)	39 (27.9)	
33 mm	25 (20.0)	40 (34.2)	43 (30.7)	
LAA ostia diameter, mm	22.4 ± 3.4	23.8 ± 3.7	23.3 ± 3.5	0.006
Anticoagulation (*n*, %)				0.543
Warfarin	4 (3.2)	6 (5.2)	3 (2.1)	
Dabigatran	29 (23.2)	27 (23.1)	26 (18.6)	
Rivaroxaban	93 (74.4)	85 (72.6)	112 (80.0)	

BMI, body mass index; CAD, coronary artery disease; NT-proBNP, N-terminal pro b-type natriuretic peptide; ALT, alanine transaminase; AST, aspartate aminotransferase; SCr, serum creatinine; TC, total cholesterol; TG, triglycerides; LDL-C, low-density lipoprotein cholesterol; LAD, left atrium diameter; LVEF, left ventricular ejection fraction; LAA, left atrial appendage.

### 3.2 Peri-procedural complications

As shown in Table 2, the overall incidence of peri-procedural complications was low across all groups. No adverse events were reported in the CBA combined with LAAO or LAAO-alone groups. In contrast, four adverse events (2.9%) occurred in the RFCA combined with the LAAO group, including 1 case of severe bleeding and 3 cases of pericardial effusion. No embolization or pseudoaneurysm was observed in any of the groups. There were no statistically significant differences in the overall incidence of complications among the groups (*p* = 0.347).

**Table 2:** Peri-procedural complications.

Variable	CBA combined with LAAO *n* = 125)	LAAO alone (*n* = 117)	RFCA combined with LAAO (*n* = 140)	*P* value
Total, (*n*, %)	0	0	4 (2.9)	0.347
Major bleeding, (*n*, %)	0	0	1 (0.7)	
Pericardial effusion, (*n*, %)	0	0	3 (2.1)	
embolism, (*n*, %)	0	0	0	
Pseudoaneurysm, (*n*, %)	0	0	0	

### 3.3 Imaging results 3-month post-procedure

**Figure 3:**
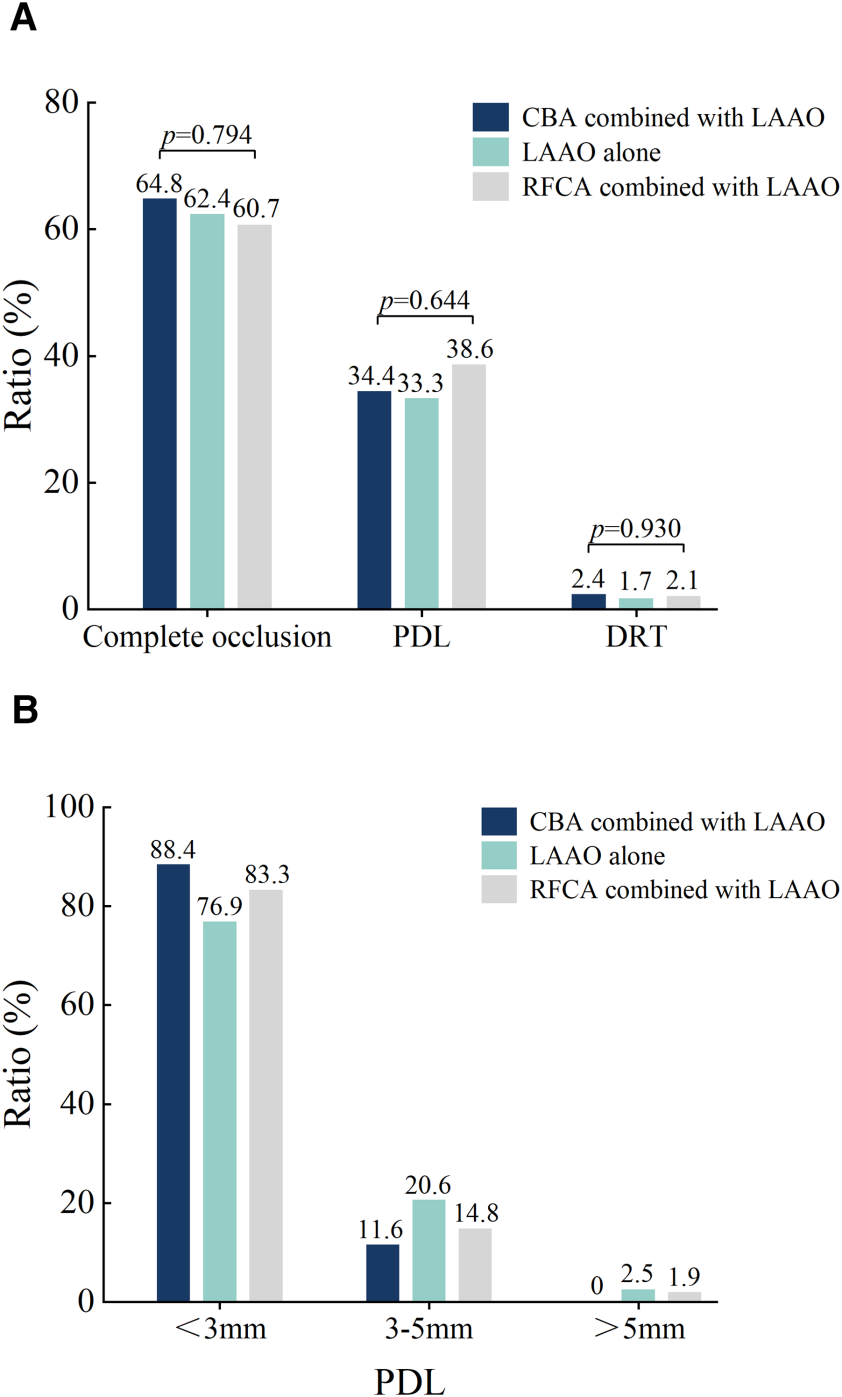
Imaging results 3-month post-procedure. **(A)** The ratios of DRT, Complete occlusion, and PDL for the three different procedures. **(B)** Comparison of PDL among three different procedures after three months. DRT, device-related thrombus; PDL, peri-device leak; CBA, cryoballoon ablation; RFCA, radiofrequency ablation; LAAO, left atrial appendage occlusion.

The imaging results at 3 months post-procedure are shown in Figure 3. The overall incidence of DRT was 3.2% across all patients. Among the groups, the incidence of DRT was 2.4% in the CBA combined with LAAO group, 1.7% in the LAAO-alone group, and 2.1% in the RFCA combined with LAAO group, with no statistically significant differences (*p* = 0.930).

Complete occlusion was achieved in 62.7% of the overall cohort. Specifically, complete occlusion rates were 64.8% in the CBA combined with the LAAO group, 62.4% in the LAAO-alone group, and 60.7% in the RFCA combined with the LAAO group, showing no significant differences among the groups (*p* = 0.794). The overall incidence of PDL was 35.1%. The incidence rates of PDL by group were 34.4% in the CBA combined with LAAO group, 33.3% in the LAAO-alone group, and 38.6% in the RFCA combined with LAAO group, with no statistically significant differences (*p* = 0.644). In terms of leak size distribution, the majority of PDLs were <3 mm, with 88.4% of leaks in the CBA combined with the LAAO group, 76.9% in the LAAO-alone group, and 83.3% in the RFCA combined with the LAAO group. The proportion of leaks measuring between 3 mm and 5 mm was 11.6% in the CBA combined with the LAAO group, 20.6% in the LAAO-alone group, and 14.8% in the RFCA combined with the LAAO group. Larger leaks (>5 mm) were rare, observed in only 1 patient (2.5%) in the LAAO-alone group and 1 patient (1.9%) in the RFCA combined with LAAO group, with no such cases in the CBA combined with LAAO group.

### 3.4 Risk factor for incomplete occlusion and PDL

Tables 3, 4 show that univariate and multivariate binary logistic regression analyses were conducted to predict incomplete occlusion and PDL. Due to the small number of cases, regression analysis was not performed for DRT and trans-fabric leaks.

**Table 3:** Logistic regression analysis of risk factors affecting incomplete occlusion after LAAO.

Variable	Univariable analysis			Multivariable analysis		
	OR	95%CI	*P* value	OR	95%CI	*P* value
Gender	0.809	0.534–1.227	0.319			
Age	0.975	0.949–1.001	0.063			
BMI	0.998	0.931–1.070	0.959			
Persistent AF	0.511	0.332–0.786	0.002	0.527	0.330–0.842	0.007
Heart failure	0.657	0.419–1.033	0.069			
Hypertension	1.290	0.838–1.987	0.248			
Diabetes	0.898	0.536–1.503	0.682			
CAD	0.801	0.468–1.370	0.418			
Stroke	0.848	0.517–1.392	0.515			
Cigarette	1.597	0.933–2.732	0.088			
Alcohol drinking	0.883	0.481–1.622	0.688			
CHA_2_DS_2_-VASc	0.882	0.770–1.011	0.071			
HAS-BLED	0.891	0.718–1.106	0.295			
NT-proBNP	1.000	1.000–1.000	0.083			
ALT	0.995	0.986–1.004	0.304			
AST	0.991	0.978–1.003	0.134			
SCr	0.967	0.955–0.978	<0.001	0.968	0.956–0.979	<0.001
TC	0.938	0.764–1.152	0.541			
TG	0.922	0.857–1.147	0.911			
LDL-C	0.973	0.816–1.160	0.761			
LAD	0.931	0.887–0.977	0.003	0.955	0.906–1.007	0.086
LVEF	1.000	0.978–1.023	0.995			
Occluder size	0.915	0.859–0.974	0.006	0.961	0.820–1.128	0.629
LAA ostia diameter	0.922	0.869–0.980	0.008	0.962	0.827–1.119	0.619

BMI, body mass index; CAD, coronary artery disease; NT-proBNP, N-terminal pro b-type natriuretic peptide; ALT, alanine transaminase; AST, aspartate aminotransferase; SCr, serum creatinine; TC, total cholesterol; TG, triglycerides; LDL-C, low-density lipoprotein cholesterol; LAD, left atrium diameter; LVEF, left ventricular ejection fraction; LAA, left atrial appendage.

**Table 4:** Logistic regression analysis of risk factors affecting PDL after LAAO.

Variable	Univariable analysis			Multivariable analysis		
	OR	95%CI	*P* value	OR	95%CI	*P* value
Gender	1.099	0.722–1.673	0.660			
Age	1.015	0.989–1.043	0.262			
BMI	0.963	0.900–1.031	0.279			
Persistent AF	2.094	1.349–3.249	<0.001	2.078	1.291–3.347	0.003
Heart failure	1.453	0.922–2.292	0.107			
Hypertension	0.833	0.538–1.290	0.413			
Diabetes	1.069	0.635–1.802	0.801			
CAD	1.182	0.686–2.035	0.547			
Stroke	1.148	0.696–1.894	0.589			
Cigarette	0.691	0.403–1.183	0.178			
Alcohol drinking	1.241	0.675–2.283	0.487			
CHA_2_DS_2_-VASc	1.097	0.956–1.258	0.188			
HAS-BLED	1.175	0.943–1.463	0.151			
NT-proBNP	1.000	1.000–1.000	0.150			
ALT	1.006	0.997–1.016	0.185			
AST	1.012	0.999–1.026	0.061			
SCr	1.034	1.002–1.046	<0.001	1.033	1.021–1.046	<0.001
TC	1.053	0.855–1.296	0.628			
TG	1.013	0.875–1.174	0.862			
LDL_C	1.096	0.920–1.306	0.304			
LAD	1.071	1.021–1.123	0.005	1.039	0.986−1.096	0.155
LVEF	0.995	0.972–1.107	0.637			
Occluder size	1.080	1.014–1.150	0.017	1.004	0.855–1.180	0.959
LAA ostia diameter	1.076	1.013–1.143	0.018	1.064	0.913–1.240	0.428

BMI, body mass index; CAD, coronary artery disease; NT-proBNP, N-terminal pro b-type natriuretic peptide; ALT, alanine transaminase; AST, aspartate aminotransferase; SCr, serum creatinine; TC, total cholesterol; TG, triglycerides; LDL-C, low-density lipoprotein cholesterol; LAD, left atrium diameter; LVEF, left ventricular ejection fraction; LAA, left atrial appendage.

In the analysis of predictors for incomplete occlusion, univariate analysis revealed that persistent AF, LAD, occluder size, LAA ostia diameter and serum creatinine (SCr) were potential influencing factors for IDE. In the multivariate analysis, persistent AF (OR = 0.527, 95% CI: 0.330–0.842, *p* = 0.007), and SCr (OR = 0.968, 95% CI: 0.956–0.979, *p *< 0.001) were identified as independent predictors.

The univariate analysis of PDL showed similar results to those of incomplete occlusion. In the multivariate analysis, persistent AF (OR = 2.078, 95% CI: 1.291–3.347, *p* = 0.003), and SCr (OR = 1.033, 95% CI: 1.021–1.046, *p *< 0.001) remained independent predictors.

## 4 Discussion

This study evaluated the safety of three treatment approaches in patients with non-valvular AF: CBA combined with LAAO, RFCA combined with LAAO, and LAAO alone. The findings demonstrated that the peri-procedural safety, complete or incomplete occlusion, PDL, and DRT outcomes were comparable across all three approaches. Multivariate analysis revealed that persistent AF and elevated SCr levels were associated with incomplete occlusion and PDL.

### 4.1 Peri-procedural complications

Regarding peri-procedural complications, our study showed that the complication rates were relatively low in both the combined ablation and LAAO-alone groups. No significant differences were observed in the incidence of adverse events (e.g., pericardial tamponade, major bleeding, or pseudoaneurysm), indicating good short-term safety for all three approaches. Notably, although three cases of pericardial tamponade occurred in the RFCA combined with the LAAO group, the difference was not statistically significant. This finding was consistent with the results of Ma et al., who also reported low peri-procedural complication rates in combined procedures, further supporting the clinical safety of this approach (16). Additionally, the study by Fassini et al. (14) reported that none of the 35 patients who underwent the “one-stop” procedure experienced major SAEs during a 24-month follow-up, with only 1 case of stroke observed during a longer follow-up period. These findings suggest that the combined procedure is not only safe in the short term but also maintains good safety over long-term follow-up. These data provide further support for the clinical use of the combined ablation and LAAO procedure, although larger prospective studies are needed to confirm its long-term safety and efficacy.

### 4.2 Post-procedural anticoagulation therapy and safety

Although this study did not directly compare the efficacy of different postoperative anticoagulation strategies, current clinical guidelines recommend routine anticoagulation and antiplatelet therapy postoperatively (19). Baseline data indicate that most patients were treated with direct oral anticoagulants (DOACs) after surgery, with rivaroxaban and dabigatran used in 80% and 23.2% of cases, respectively. In comparison, warfarin was used in only 3.2% of cases. These results reflect the current clinical practice trend, where DOACs have gradually replaced warfarin due to their lower bleeding risks and the absence of the need for routine monitoring. A systematic review and network meta-analysis published in *JACC* (20) suggested that novel oral anticoagulants (NOACs) may be the optimal initial anticoagulation strategy after LAAO, as they offer a higher potential for reducing thromboembolic events and major bleeding risks. Further analysis indicated that NOACs were significantly superior to warfarin in reducing the risk of all-cause mortality (OR = 0.39).

A recent study, the OPTION trial (21), further explored the safety and efficacy of LAAO vs. OACs after ablation for AF during long-term follow-up (3 years). This study showed that the incidence of bleeding events in the LAAO group was significantly lower than that in the oral anticoagulant group (8.5% vs. 18.1%, *p* < 0.0001). However, the incidence of death from any cause, stroke, or systemic embolism was similar between the two groups (5.3% vs. 5.8%, *p* < 0.0001). These findings suggest that catheter-based AF ablation combined with LAAO was a non-inferior method for preventing stroke when compared with oral anticoagulants. In the present study, catheter-based AF ablation combined with LAAO, whether using RFCA or CBA, demonstrated both safety and effectiveness. However, this study primarily evaluated the short-term safety of different LAAO strategies.

### 4.3 Complete and incomplete occlusion

Our study revealed no significant differences in complete and incomplete occlusion rates among the three groups; however, the CBA combined with the LAAO group exhibited a higher rate of complete occlusion than the RFCA combined with LAAO and LAAO-alone groups. Complete occlusion is a crucial factor for long-term device stability and thrombus prevention, and it may be influenced by the local inflammatory response triggered by ablation. RFCA induces thermal injury, leading to coagulative necrosis of myocardial tissue and more extensive tissue disruption. In contrast, CBA utilizes cold temperatures, which preserves cellular ultrastructure, thus reducing endothelial damage and minimizing the risk of thrombus formation. This difference in ablation mechanisms may contribute to the superior occlusion outcomes observed in the CBA combined with the LAAO group (22, 23). Previous studies also supported this conclusion. For example, Khairy et al. (24) found that RFCA was more likely to induce thrombus formation compared to CBA, likely due to the broader inflammatory response and more extensive tissue injury associated with RFCA. In contrast, the relatively milder inflammatory response triggered by CBA may facilitate vascular endothelial cell proliferation and repair, thereby accelerating the endothelialization process, and, in turn, expediting complete occlusion. Additionally, previous research has demonstrated that combining AF ablation with LAAO can improve electrophysiological function and hemodynamics in AF patients (25). These mechanisms may provide a plausible explanation for the observed difference in complete occlusion rates between the CBA combined with LAAO and RFCA combined with LAAO groups in this study. However, the difference did not reach statistical significance.

Incomplete occlusion is closely associated with the risk of DRT. Studies have shown that incomplete occlusion is more prone to thrombus formation (26). Although no significant differences in DRT incidence were observed among the three groups in this study, the higher complete occlusion rate in the CBA combined with the LAAO group suggests a potential for lower long-term thrombus formation risk. Both Dukkipati et al. (27) and Fauchier et al. (28) reported that patients with DRT had significantly higher rates of ischemic stroke or systemic embolism. Among AF patients undergoing percutaneous LAAO, the stroke rate was 15.4% in the DRT group compared to 3.2% in the non-DRT group. However, Lempereur et al. (29) found no significant association between different types of occlusion devices and cerebrovascular events despite the increased risk of DRT. Therefore, the specific mechanisms by which DRT affects long-term outcomes remain to be elucidated. Due to the limited number of DRT events in this study, it was impossible to conduct a comprehensive statistical analysis. Therefore, further investigation into the risk factors associated with DRT was not pursued.

Univariate analysis in this study identified persistent AF, LAD, LAA ostium size, and occluder size as risk factors significantly associated with incomplete occlusion, consistent with previous literature (30–32). The multivariate analysis identified persistent AF and SCr levels as independent predictors of incomplete occlusion. Persistent AF often leads to pathological remodelling and hemodynamic alterations in the atria, which can negatively impact complete occlusion. SCr, a key indicator of renal function, also reflects the patient's metabolic state and systemic inflammatory response. Elevated SCr levels may increase the risk of incomplete occlusion through several mechanisms. First, elevated SCr levels can indicate mild to moderate renal impairment, even if the condition has not yet reached the clinical threshold for diagnosing “renal insufficiency”. Renal impairment is frequently associated with endothelial dysfunction (33), which can reduce the endothelial cell repair and regeneration capacity, thereby delaying or hindering the complete occlusion process of the occlusion device. Second, systemic inflammation often accompanies renal impairment, which may further disrupt endothelial cell function and compromise the complete occlusion process (34).

### 4.4 Peri-device leak

PDL is a common complication following LAAO and can increase the risk of thrombus formation if it persists (35). In this study, the incidence of PDL was 33.3% in the LAAO-alone group, lower than in the CBA combined with LAAO group (34.4%) and RFCA combined with LAAO group (38.6%). However, the differences among the groups were not statistically significant (*p* = 0.644). Clinically, PDL is a critical concern and may be influenced by several factors, including LAA anatomy, procedural technique, operator experience, and device positioning (36). Zhao et al. (37) demonstrated that the maximum diameter of the LAA orifice can independently predict the occurrence of postoperative PDL following LAAO, which aligns with the findings of our study.

Multivariate analysis further revealed that persistent AF and elevated SCr levels are independent risk factors for PDL. As discussed previously, elevated SCr reflects impaired renal function and systemic inflammation, which may hinder the healing process of the occluder and contribute to the development of PDL. A single-centre retrospective study involving 172 patients by Chen et al. (38) found that patients without PDL had a faster rate of endothelialization. In contrast, PDL was identified as an independent risk factor for delayed endothelialization. Saw et al. (39) highlighted several mechanisms contributing to PDL, including off-axis device placement, misalignment between the LAA landing zone and the occluder, insufficient expansion of the LAA landing zone, resulting in gaps around the device, and incomplete device endothelialization (IDE). The presence of IDE and PDL will ultimately lead to incomplete occlusion.

Given the significant overlap in risk factors for PDL and incomplete occlusion, preoperative evaluation of SCr levels could be an important predictor of postoperative complications. For patients with elevated SCr, close postoperative monitoring of device healing and adjustments to anticoagulant therapy is recommended based on the patient's metabolic status. This approach may mitigate potential risks and improve long-term outcomes by promoting more effective device integration and complete occlusion.

## 5 Conclusion

The results of this study demonstrated no significant differences in peri-procedural complications, Complete or incomplete occlusion, PDL, or DRT rates among the three surgical approaches. However, persistent AF and elevated SCr levels were identified as independent risk factors for PDL and incomplete occlusion. These findings suggest that individualized treatment strategies should be developed for high-risk AF patients based on their clinical characteristics to optimize both peri-procedural and post-procedural outcomes.

## 6 Limitations

This study has several limitations. First, it was a single-centre, retrospective observational study. Despite using multivariate analysis to minimize the impact of confounding factors, selection bias may still be present. Additionally, the follow-up period was relatively short (only 3 months post-procedure), limiting the ability to assess long-term clinical outcomes. Another potential limitation is the lack of detailed classification within incomplete occlusion (including PDL, Trans-fabric leak, and both), which may have influenced the results to some extent.

## Data Availability

The raw data supporting the conclusions of this article will be made available by the authors, without undue reservation.
